# LINC00261 Is Differentially Expressed in Pancreatic Cancer Subtypes and Regulates a Pro-Epithelial Cell Identity

**DOI:** 10.3390/cancers12051227

**Published:** 2020-05-13

**Authors:** Agnes Dorn, Markus Glaß, Carolin T. Neu, Beate Heydel, Stefan Hüttelmaier, Tony Gutschner, Monika Haemmerle

**Affiliations:** 1 Institute of Pathology, Section for Experimental Pathology, Medical Faculty, Martin-Luther University Halle-Wittenberg, 06120 Halle/Saale, Germany; agnes.dorn@uk-halle.de (A.D.); carolin.neu@uk-halle.de (C.T.N.); beate.heydel@uk-halle.de (B.H.); 2 Institute of Molecular Medicine, Section for Cell Biology, Medical Faculty, Martin-Luther University Halle-Wittenberg, 06120 Halle/Saale, Germany; markus.glass@medizin.uni-halle.de (M.G.); stefan.huettelmaier@medizin.uni-halle.de (S.H.); 3 Junior Research Group ‘RNA biology and Pathogenesis’, Medical Faculty, Martin-Luther University Halle-Wittenberg, 06120 Halle/Saale, Germany

**Keywords:** PDAC, lncRNA, LINC00261, EMT, TGFβ, CDH1, FOXA2

## Abstract

Pancreatic adenocarcinoma (PDAC) is one of the major causes of cancer-associated deaths worldwide, with a dismal prognosis that has not significantly changed over the last decades. Transcriptional analysis has provided valuable insights into pancreatic tumorigenesis. Specifically, pancreatic cancer subtypes were identified, characterized by specific mutations and gene expression changes associated with differences in patient survival. In addition to differentially regulated mRNAs, non-coding RNAs, including long non-coding RNAs (lncRNAs), were shown to have subtype-specific expression patterns. Hence, we aimed to characterize prognostic lncRNAs with deregulated expression in the squamous subtype of PDAC, which has the worst prognosis. Extensive in silico analyses followed by in vitro experiments identified long intergenic non-coding RNA 261 (LINC00261) as a downregulated lncRNA in the squamous subtype of PDAC, which is generally associated with transforming growth factor β (TGFβ) signaling in human cancer cells. Its genomic neighbor, the transcription factor forkhead box protein A2 (FOXA2), regulated LINC00261 expression by direct binding of the LINC00261 promoter. CRISPR-mediated knockdown and promoter knockout validated the importance of LINC00261 in TGFβ-mediated epithelial–mesenchymal transition (EMT) and established the epithelial marker E-cadherin, an important cell adhesion protein, as a downstream target of LINC00261. Consequently, depletion of LINC00261 enhanced motility and invasiveness of PANC-1 cells in vitro. Altogether, our data suggest that LINC00261 is an important tumor-suppressive lncRNA in PDAC that is involved in maintaining a pro-epithelial state associated with favorable disease outcome.

## 1. Introduction

Pancreatic adenocarcinoma (PDAC) is currently the fourth most common cause of cancer-related death in developed countries. Due to the lack of symptoms and diagnosis in late stages and the overall high resistance to currently available treatment options, the number of deaths is estimated to increase significantly over the next 20 years [1,2]. Integrated genomic analyses have identified main disease subtypes that are associated with differences in overall survival and therapy response. Bailey et al. used bulk tumor tissue and defined four molecular subtypes, which are characterized by a specific set of mutations and gene expression programs [3]. Actual and virtual microdissection of the tumor epithelium identified three and two subtypes, respectively [4,5]. Similarly, Poleo et al. confirmed two subtypes that had been published earlier in high cellularity samples and after removing transcripts native to the normal pancreas [6]. Interestingly, one subtype is common to all these methodologically different analyses. The “squamous”, “basal-like”, or “quasi-mesenchymal” subtype is characterized by poor prognosis and deregulated expression of genes, which are important for epithelial–mesenchymal transition (EMT) [7]. During EMT, epithelial cells lose their cell-specific phenotype and acquire characteristics of invasive mesenchymal cells [8]. EMT is associated with expression changes of several genes, including the downregulation of epithelial markers, such as E-cadherin (CDH1), and the upregulation of mesenchymal markers, such as N-cadherin (CDH2), vimentin (VIM), and fibronectin 1 (FN1) [9]. Notably, it has been shown that EMT plays a major role in the metastatic process of PDAC [10]. In addition to its role in cancer, EMT and its reversed process, mesenchymal-to-epithelial transition (MET), are fundamental processes in embryonic development and organ differentiation. EMT is stimulated by the transforming growth factor β (TGFβ) via binding to TGFβ receptors and downstream activation of SMAD (Mothers against DPP Homolog) proteins that translocate into the nucleus and regulate cell type-specific expression of EMT-promoting genes [11]. Fine-tuning of this pathway is maintained by transcriptional and post-transcriptional mechanisms, by epigenetic modifications, and by non-coding RNA-mediated regulation, whereby both microRNAs (miRNAs) and long non-coding RNAs (lncRNAs) are involved [12]. LncRNAs are defined as endogenous RNAs that consist of more than 200 nucleotides. In recent years, an enormous number of lncRNAs have been identified in the human genome. While the precise function for the majority of these molecules is largely unknown, several regulatory mechanisms of lncRNAs in different cellular processes have been described [13]. These mechanisms include gene expression regulation at different levels, such as transcriptional and post-transcriptional control or chromatin modification. Importantly, deregulation of lncRNAs has been observed in different types of cancer, implying that they are crucial regulators and potential diagnostic biomarkers in cancer [14]. Intriguingly, differences in lncRNA expression have also been associated with different PDAC subtypes, highlighting their roles in PDAC progression, therapy response, and patient survival [15]. Given the relevance of EMT for PDAC progression and metastasis, as well as its key role in defining the squamous subtype of this disease, our aim was to identify and characterize the function of lncRNA candidates that might be involved in shaping the molecular landscape of this highly aggressive subtype [3]. Extensive in silico analysis identified one promising lncRNA candidate, long intergenic non-coding RNA 261 (LINC00261), which was strongly downregulated in the squamous subtype, inversely correlated with tumor stage and tumor grade, and associated with favorable disease outcome. This suggested a tumor-suppressive nature of this lncRNA, which was supported by analyses in other cancer types, including hepatocellular carcinoma, gastric, and lung cancer [16,17,18]. Aiming to study the regulation and function of LINC00261 in PDAC, we combined bioinformatical analysis of publicly available datasets and RNA sequencing studies to identify pathways associated with this lncRNA. Consistent with earlier reports on other tumor types, gene set enrichment analysis (GSEA) revealed a negative correlation of LINC00261 expression with the hallmark gene set “epithelial–mesenchymal transition”. Matching in silico data analysis, we found a strong downregulation of E-cadherin at the transcriptional and protein levels after depletion of LINC00261 using CRISPR interference (CRISPRi), as well as in three different LINC00261 promoter knockout clones. In line with this, LINC00261 depletion led to significantly increased pancreatic cancer cell invasion and migration. Moreover, we found that the transcription factor forkhead box protein A2 (FOXA2) could bind to the promoter of LINC00261, thereby regulating its expression.

In summary, our data suggest that LINC00261 is an important tumor-suppressive lncRNA in PDAC, which shows a subtype-specific expression pattern and an association with EMT. We propose that LINC00261 might help to establish a pro-epithelial cellular phenotype and likely a state of increased differentiation, leading to better overall survival in patients with LINC00261^high^ tumors.

## 2. Results

### 2.1. LINC00261 is Downregulated in PDAC and its Expression Correlates with PDAC Subtypes, Stage, Grade, and Patient Survival

**Figure 1:**
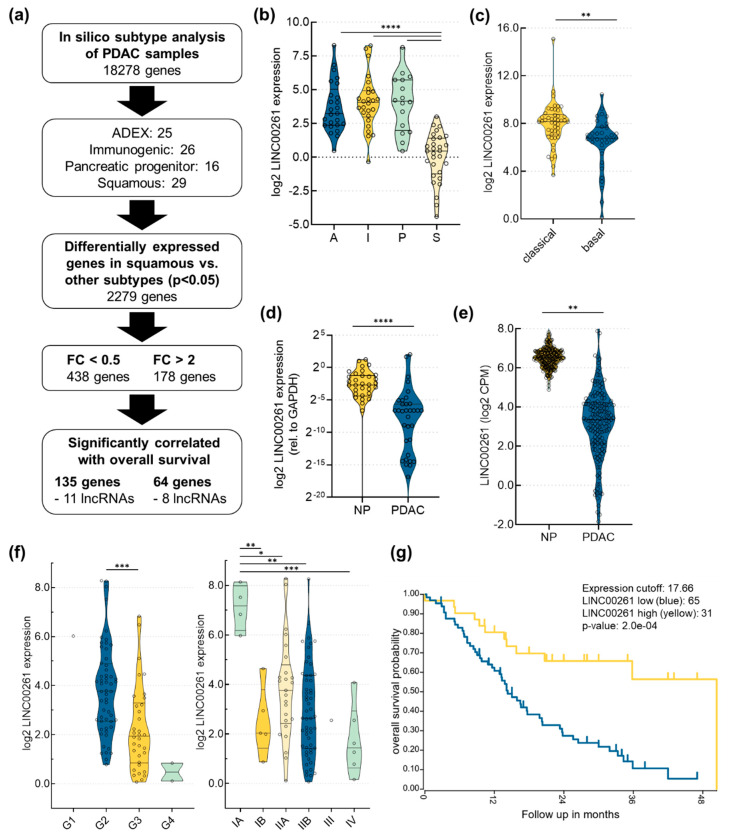
Analysis of long intergenic non-coding RNA 261 (LINC00261) expression in pancreatic adenocarcinoma (PDAC). (**a**) Flow chart that outlines the identification of candidate long non-coding RNAs (lncRNAs) important for PDAC progression and patient survival; ADEX, aberrantly differentiated endocrine exocrine; FC, fold change. (**b**) Analysis of the Bailey PDAC dataset revealed a significant downregulation of LINC00261 expression in the squamous (S) compared to the pancreatic progenitor (P), immunogenic (I), and ADEX (A) subtypes (**** *p* < 0.0001, one-way ANOVA). (**c**) Analysis of the pancreatic adenocarcinoma dataset from The Cancer Genome Atlas (TCGA) according to Moffitt’s classification highlighted significant downregulation of LINC00261 expression in the basal-like compared to the classical subtype (** *p* < 0.01, unpaired t-test). (**d**,**e**) Analysis of LINC00261 expression in 34 normal pancreatic (NP) tissues, 42 PDAC tissues (**d**), and in publicly available TCGA and Genotype-Tissue Expression (GTEx) datasets (**e**) (normal pancreas: *n* = 177, PDAC: *n* = 248) showed significantly lower LINC00261 expression in pancreas adenocarcinoma compared to normal pancreas (** *p* < 0.01, **** *p* < 0.0001, Mann–Whitney U test). (**f**) LINC00261 expression is significantly lower in high grade (G1: *n* = 1, G2: *n* = 56, G3: *n* = 34, G4: *n* = 2) and high-stage tumors (IA: *n* = 4, IB: *n* = 5, IIA: *n* = 25, IIB: *n* = 55, III: *n* = 1, IV: *n* = 6); * *p* < 0.05, ** *p* < 0.01, *** *p* < 0.001, **** *p* < 0.0001, one-way ANOVA. (**g**) Survival analysis for PDAC patients with low LINC00261 (blue line, *n* = 65) versus high LINC00261 (yellow line, *n* = 31) expression (Bailey dataset, http://r2.amc.nl, Log rank test).

In order to identify lncRNAs associated with PDAC subtypes, we performed in silico analyses focusing on the publicly available PDAC dataset of Bailey et al. [3], which defined four disease subtypes by RNA expression analysis: squamous, pancreatic progenitor, immunogenic, and aberrantly differentiated endocrine exocrine (ADEX) (Figure 1a).

Firstly, we applied the previously published non-negative matrix factorization (NMF) algorithm [19] to the International Cancer Genome Consortium (ICGC) PDAC data and identified these four described disease subtypes. We could assign 25 samples to the ADEX subtype, 26 samples to the immunogenic subtype, 16 samples to the pancreatic progenitor subtype, and 29 samples to the squamous subtype. Patients with tumors characterized by the squamous subtype were shown to have significantly worse overall survival compared to patients with tumors of all other disease subtypes [3]. In order to identify potential disease driving mechanisms responsible for dismal patient prognosis, we focused on differently expressed RNAs in the squamous subtype versus all other subtypes, which led to the identification of 2279 RNAs (*p* < 0.05). By applying an absolute fold change (FC) cut-off of 2.0 and 0.5, 438 genes were found to be downregulated, whereas 178 genes were upregulated in the squamous subtype. Next, we leveraged the R2 Genomics Analysis and Visualization Platform (http://r2.amc.nl) to assess the prognostic relevance of all 616 genes on overall survival, using the median expression of each gene as a cut-off to define high and low expression groups. This analysis identified 199 genes as being significantly associated with disease survival, including 19 lncRNAs (Table S1). By applying these stepwise analyses, LINC00261 was identified as the lncRNA with the most significant difference between the identified groups, showing a strong downregulation in the squamous subtype compared to all other published subtypes (Figure 1b). Methylation and gene expression analysis of squamous tumors showed hypermethylation and downregulation of genes important for determination of endodermal cell fate, for example pancreatic and duodenal homeobox 1 (PDX1), motor neuron and pancreas homeobox 1 (MNX1), and GATA binding protein 6 (GATA6). In contrast, squamous tumors were enriched for activated epidermal growth factor (EGF) signaling associated with hypomethylation and upregulation of epidermal growth factor receptor (EGFR), as well as upregulation of key factors involved in metastasis, including lysyl oxidase (LOX) [3]. Moreover, yes-associated protein 1 (YAP1) expression was very recently shown to be crucial for maintenance of the squamous subtype in pancreatic cancer [20]. Similar gene expression changes were found in a study using patient-derived tumor xenografts [21]. Intriguingly, we found that LINC00261 expression was positively correlated with the expression of genes that are crucial for endodermal differentiation, and was negatively correlated with expression of EGFR, LOX, and YAP1 (Figure S1). In addition, LINC00261 expression was also significantly downregulated in the basal-like PDAC subtype defined by Moffitt et al. [5], which closely resembles the squamous subtype [3] (Figure 1c). Furthermore, own RNA expression analysis of formalin-fixed paraffin-embedded normal pancreas (NP) and PDAC tissue (Figure 1d, Table S2), as well as expression analysis of publicly available data provided by The Cancer Genome Atlas [15] and the Genotype-Tissue Expression (GTEx) platform ([22], Figure 1e), showed that LINC00261 expression was significantly lower in PDAC compared to normal pancreatic tissue. Further in-depth analysis of LINC00261 revealed an inverse correlation of its expression with tumor grade and tumor stage (Figure 1f). Intriguingly, high expression of LINC00261 was associated with significantly better overall survival in PDAC patients using the Bailey dataset (Figure 1g). Similar results were found for TCGA data of PDAC, as well as lung adenocarcinoma using Gene Expression Profiling Interactive Analysis (GEPIA [23]). Altogether, these results suggest that LINC00261 is a tumor-suppressive lncRNA in pancreatic and lung adenocarcinoma associated with tumor stage and grade, as well as favorable disease outcome.

### 2.2. LINC00261 is Associated with EMT and is Regulated by its Genomic Neighbour FOXA2

**Figure 2:**
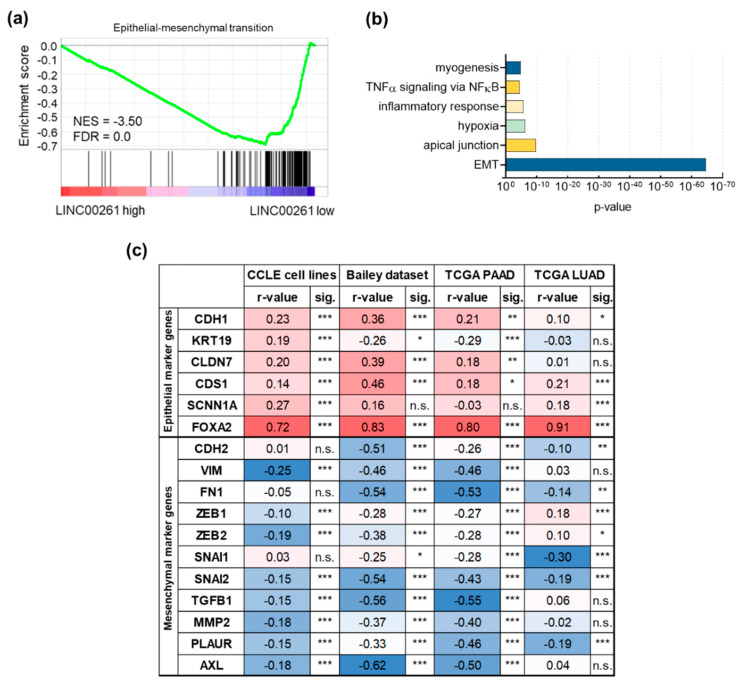
Association of long intergenic non-coding RNA 261 (LINC00261) with epithelial–mesenchymal transition (EMT). (**a**) Gene set enrichment analysis (GSEA) of PDAC samples with low (*n* = 48) versus high LINC00261 expression (*n* = 48). Median LINC00261 expression was used as a cut-off; NES, normalized enrichment score; FDR, false discovery rate. (**b**) GSEA analysis of genes that were significantly inversely correlated with LINC00261 in the pancreatic adenocarcinoma (PDAC) dataset of Bailey et al. identified EMT as the highest enriched hallmark gene set. (**c**) Correlation of selected epithelial and mesenchymal marker genes with LINC00261 expression (* *p* < 0.05, ** *p* < 0.01, *** *p* < 0.001). Note: CDH1, E-cadherin; KRT19, keratin 19; CLDN7, claudin 7; CDS1, CDP-diacylglycerol synthase 1; SCNN1A, sodium channel epithelial 1 subunit alpha; FOXA2, forkhead box A2; CDH2, N-cadherin; VIM, vimentin; FN1, fibronectin 1; ZEB1, zinc finger E-box binding homeobox 1; ZEB2, zinc finger E-box binding homeobox 2; SNAI1, snail family transcriptional repressor 1; SNAI2, snail family transcriptional repressor 2; TGFB1, transforming growth factor beta 1; MMP2, matrix metallopeptidase 2; PLAUR, plasminogen activator, urokinase receptor; AXL, AXL receptor tyrosine kinase.

In order to identify pathways associated with deregulated LINC00261 expression, we performed gene set enrichment analysis (GSEA, [24,25]) of differentially expressed genes in PDAC samples of the Bailey dataset, using LINC00261 expression level as a discriminator to define LINC00261^low^ and LINC00261^high^ sample groups. Here, median LINC00261 expression was used as a cut-off to assign samples to both groups. Importantly, GSEA revealed a significant enrichment of an EMT signature in LINC00261^low^ tumors (Figure 2a). Additionally, the significant correlation of GSEA of genes with LINC00261 expression in this dataset confirmed this result (Figure 2b). Moreover, selected epithelial and mesenchymal marker genes [26] showed significant positive and negative correlations with LINC00261 expression, respectively, in the Cancer Cell Line Encyclopedia (CCLE) dataset [27,28], two different PDAC sample sets [3,15], and the TCGA lung adenocarcinoma (LUAD) dataset [29] (Figure 2c).

**Figure 3:**
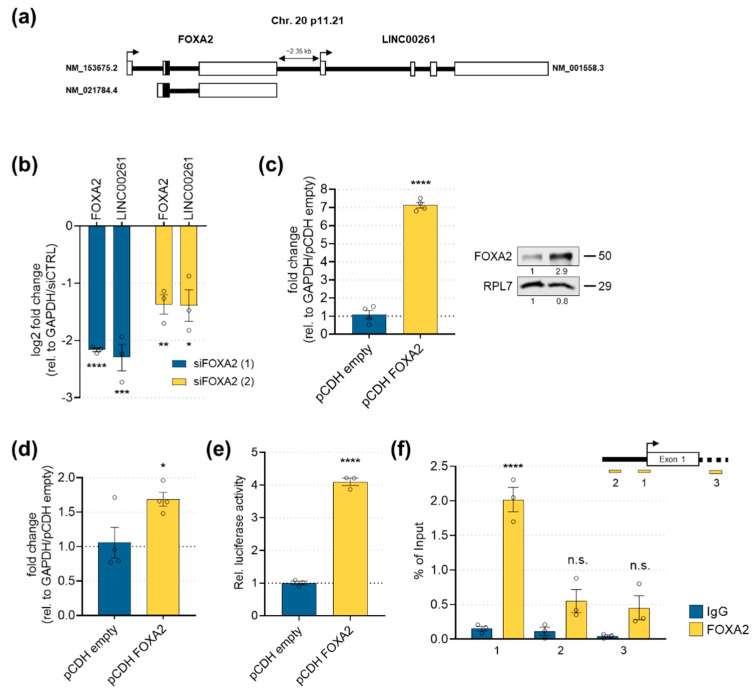
LINC00261 regulation by its genomic neighbor FOXA2. (**a**) The genomic loci of LINC00261 and FOXA2 on chromosome 20. (**b**) The siRNA-mediated knockdown of FOXA2 significantly downregulated FOXA2 and LINC00261 RNA levels (* *p* < 0.05, ** *p* < 0.01, *** *p* < 0.001, **** *p* < 0.0001, one-way ANOVA). (**c**) Stable overexpression of FOXA2 in PANC-1 was achieved at RNA (left panel; glyceraldehyde-3-phosphate dehydrogenase (GAPDH) was used as the reference gene) and protein levels (right panel; ribosomal protein L7 (RPL7) was used as a loading control; **** *p* < 0.0001, unpaired t-test). (**d**) LINC00261 expression levels in control or FOXA2 overexpressing PANC-1 cells (* *p* < 0.05, unpaired t-test). (**e**) Luciferase activity of a LINC00261 promoter reporter after stable FOXA2 or empty control vector overexpression in PANC-1 cells (**** *p* < 0.0001, unpaired t-test). (**f**) Chromatin immunoprecipitation (ChIP) followed by qPCR analysis using primers located upstream (1,2) and downstream (3) of the LINC00261 transcriptional start site (upper panel) confirmed binding of FOXA2 to the LINC00261 promoter region (lower panel, **** *p* < 0.0001, unpaired t-test).

Interestingly, the transcription factor FOXA2 is a genomic neighbor of LINC00261 (Figure 3a), which was positively correlated (*r* = 0.72–0.91) with LINC00261 expression in all datasets analyzed. Importantly, FOXA2 has been described as an epithelial marker and inhibitor of EMT in several tumor types [17,30,31,32]. Moreover, FOXA2 was shown to be a crucial denominator of differentiation of human pluripotent stem cells into pancreatic progenitors [33]. In line with its high correlation to LINC00261, FOXA2 showed a similar expression pattern across different PDAC subtypes of the Bailey dataset (Figure S2). To investigate whether FOXA2 could regulate LINC00261 expression in pancreatic cancer cells, we manipulated FOXA2 levels in PANC-1 cells. Knockdown of FOXA2 using two different small interfering RNAs (siRNAs) led to a strong decrease of LINC00261 transcript levels (Figure 3b). To analyze a direct transcriptional regulation by FOXA2, we stably overexpressed FOXA2 in PANC-1 cells and subsequently performed luciferase assays to determine LINC00261 promoter activity. Here, moderate overexpression of FOXA2 mRNA and protein in PANC-1 cells (Figure 3c) led to a significant increase in LINC00261 RNA expression (Figure 3d) and LINC00261 promoter activity (Figure 3e). Finally, we investigated the physical interaction of FOXA2 with the LINC00261 promoter using a chromatin immunoprecipitation–quantitative polymerase chain reaction (ChIP-qPCR) experiment. The ChIP-qPCR analysis demonstrated a strong binding of FOXA2 to the promoter region of LINC00261, whereas neither binding of FOXA2 to the upstream proximal nor downstream intragenic regions of LINC00261 could be detected (Figure 3f).

These results suggest a direct regulation by and tight interconnection of LINC00261 with its genomic neighbor FOXA2, which is strongly supported by previous results in lung adenocarcinoma cell lines [34].

### 2.3. LINC00261 is Downregulated by TGFβ in TGFβ-Responsive Cell Lines

**Figure 4:**
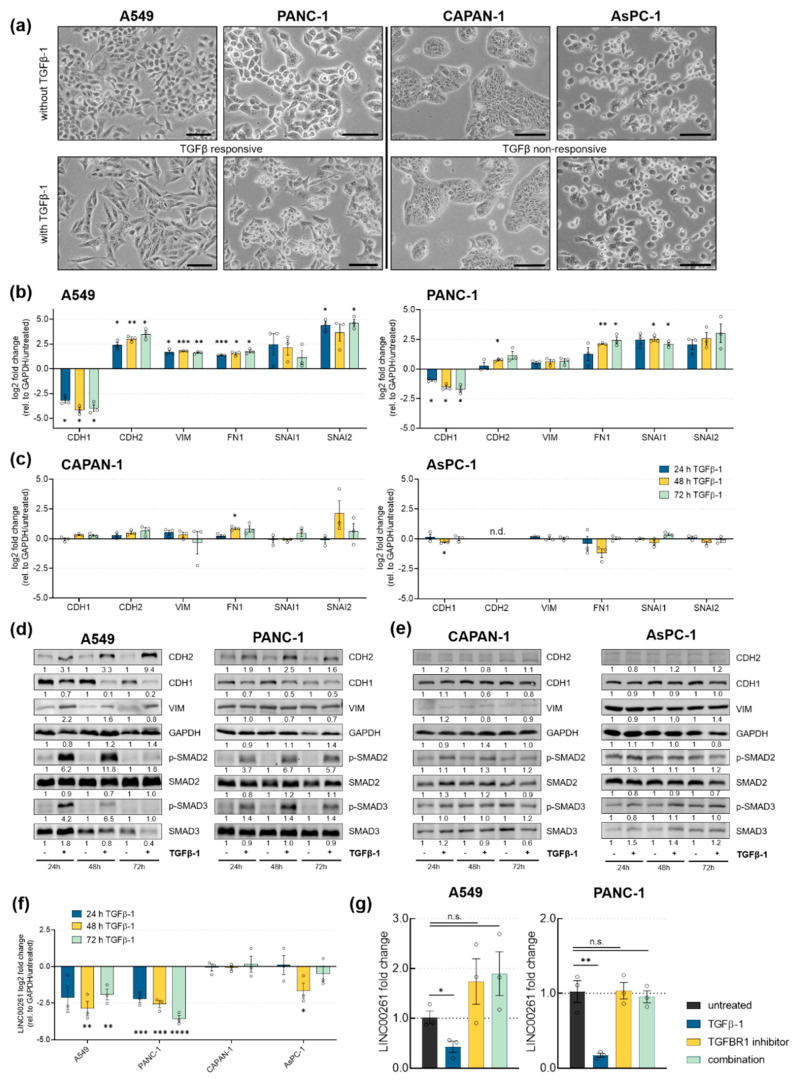
Transforming growth factor β (TGFβ) downregulates LINC00261 expression in cell lines that show TGFβ-induced EMT. (**a**) Brightfield images of untreated and TGFβ-1 treated A549, PANC-1 (TGFβ-responsive), AsPC-1, and CAPAN-1 cells (TGFβ-non-responsive). Scale bar = 100 µm. Analysis of mRNA (**b**,**c**) and protein levels (**d**,**e**) of genes associated with EMT was done by qRT-PCR and Western Blot, respectively (* *p* < 0.05, ** *p* < 0.01, *** *p* < 0.001, two-way ANOVA). (**f**) LINC00261 expression in TGFβ-responsive and non-responsive cells using qRT-PCR (* *p* < 0.05, ** *p* < 0.01, *** *p* < 0.001, **** *p* < 0.0001, unpaired t-test). (**g**) LINC00261 regulation in A549 and PANC-1 cells treated with TGFβ-1, TGFBR1 inhibitor (RepSox), or both after 72 h, measured by qRT-PCR (* *p* < 0.05, ** *p* < 0.01, unpaired *t*-test).

Our in silico analysis presented above, as well as previously published data on LINC00261 in gastric [35], hepatocellular [18], and lung carcinoma [17], suggested an association of LINC00261 with TGFβ signaling and EMT. To validate these findings and to analyze this connection further, we stimulated three pancreatic cancer cell lines and A549 lung adenocarcinoma cells with TGFβ for 24, 48, and 72 h. TGFβ treatment caused EMT-like morphological changes in A549 and PANC-1 cells, whereas AsPC-1 and CAPAN-1 failed to exhibit changes in morphology upon cytokine stimulation (Figure 4a). In line with these morphological changes, only the TGFβ-responsive cell lines A549 and PANC-1 also showed expression changes of EMT-associated genes, both at RNA and protein levels (Figure 4b,d). Specifically, in both cell lines TGFβ stimulation reduced the expression of the epithelial marker E-cadherin (CDH1) and led to an upregulation of mesenchymal genes including, N-cadherin (CDH2), vimentin (VIM), fibronectin 1 (FN1), snail (SNAI1), and slug (SNAI2). Additionally, in both cell lines phosphorylation of SMAD2 and SMAD3 proteins was induced, as these are important mediators of TGFβ signaling inside the cell (Figure 4d). In contrast, AsPC-1 and CAPAN-1 failed to respond to TGFβ, not only at the morphological (Figure 4a) level, but also at the molecular level (Figure 4c,e). Most notably, none of the used cell lines exhibited mutations in the major receptors for TGFβ, namely TGFBR1 and TGFBR2. Concordant with the in silico data suggesting LINC00261 association with EMT, only TGFβ-responsive lung and pancreatic cancer cell lines significantly downregulated LINC00261 expression upon exposure to TGFβ-1 for 24, 48, and 72 h (Figure 4f). Similar to LINC00261, FOXA2 is downregulated after TGFβ-1 treatment in responsive cells, but not in non-responsive cells (Figure S3). Moreover, FOXA2 binding to the LINC00261 promoter was reduced under TGFβ treatment conditions (Figure S4). To investigate whether TGFβ-mediated downregulation of LINC00261 was dependent on TGFBR1 activity, we treated A549 and PANC-1 cells with the potent and selective TGFBR1 inhibitor RepSox [36]. Indeed, downregulation of LINC00261 after 72 h by TGFβ-1 in both cell lines was completely abolished by addition of RepSox (Figure 4g). These results further support the idea that LINC00261 is a TGFβ-regulated lncRNA in TGFβ-responsive cells that show EMT-associated morphological changes, dysregulation of EMT-associated cadherins and transcription factors following classical, TGFBR1-dependent pathway activation.

### 2.4. Downregulation of LINC00261 Expression with Two Different CRISPR-Based Systems Leads to EMT

**Figure 5:**
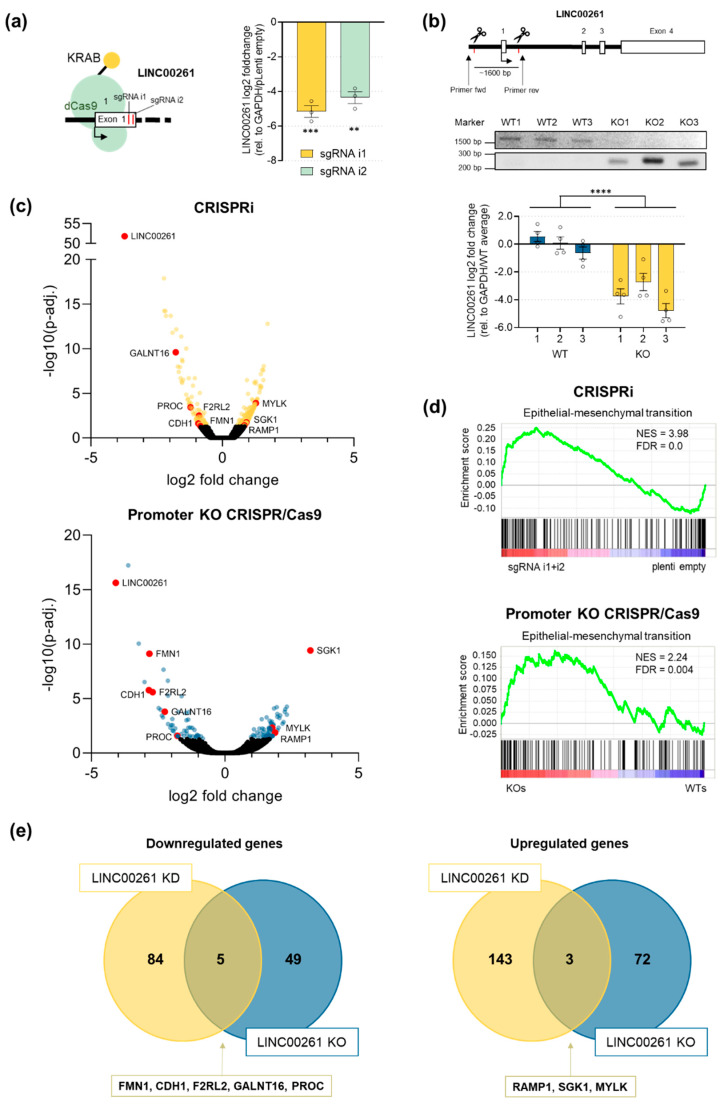
RNA-seq analysis revealed an enrichment of the EMT gene set in LINC00621^low^ PANC-1 cells. (**a**) Schema of CRISPR interference (CRISPRi)-mediated targeting of LINC00261 and its expression levels in PANC-1 cells measured by qRT-PCR (** *p* < 0.01, *** *p* < 0.001, one-way ANOVA). (**b**) Schema of CRISPR/Cas9-mediated knockout of the promoter region of LINC00261 using two sgRNAs. Cutting of both sgRNAs led to the removal of a genomic fragment of ~1600 bp. PCR and gel electrophoresis using the indicated primers resulted in a ~1600 bp product in wild-type clones and a ~250 bp product in knockout clones (**** *p* < 0.0001, two-way ANOVA). (**c**) Volcano plots of RNA-seq analyses of differently expressed genes of CRISPRi- and CRISPR/Cas9-mediated knockdown of LINC00261. Significantly deregulated genes (adjusted *p*-value (*p*-adj.) < 0.05) are highlighted. Genes found to be deregulated in both cell systems are labelled in red. (**d**) GSEA analysis of RNA sequencing data revealed a significant enrichment of the EMT gene set in LINC00261-depleted cells in both knockdown systems. (**e**) Venn diagrams show the intersection of significantly (*p*-adj. < 0.05) downregulated or upregulated genes due to LINC00261 knockdown by CRISPRi or CRISPR/Cas9 systems.

To further characterize the function of LINC00261 in PDAC, specifically in the context of EMT, we established two CRISPR-based knockdown systems of LINC00261 in PANC-1 cells. First, we applied an CRISPR interference (CRISPRi) approach [37] using two independent LINC00261-specific single-guide RNAs (sgRNAs). Both sgRNAs (i1,i2) were stably introduced into dCas9-KRAB expressing PANC-1 cells and efficiently reduced the level of LINC00261, leaving a remaining LINC00261 expression of only 5–6% in the cells (Figure 5a). In a second approach, we utilized the standard CRISPR/Cas9 system [38] to delete the potential LINC00261 promoter (~1600 bp) by applying two sgRNAs. Fluorescence-activated cell sorting was used to generate single cell clones after transient transfection of Cas9 and the two sgRNAs. Individual clones were expanded and genomic DNA was isolated to perform PCR-based screening for the presence of a ~250 bp fragment that was only detectable in promoter deleted clones (Figure 5b, upper panel). Subsequently, gene expression was analyzed by qRT-PCR, showing a strong downregulation of LINC00261 in respective promoter knockout clones compared to wild-type clones. In total, this CRISPR/Cas9-mediated targeting strategy allowed us to isolate three wild-type (WT) and three promoter knockout (KO) clones (Figure 5b, lower panel). Since both knockdown strategies (CRISPRi vs. CRISPR/Cas9) have their advantages and disadvantages, we performed our subsequent downstream analysis using both systems in parallel and focusing on the overlap of the two to decipher the functional relevance of LINC00261 in PANC-1 cells. To this end, we initially performed a comprehensive gene expression analysis using both LINC00261-depleted cell systems. First, RNA sequencing was performed in biological triplicates to identify differentially regulated genes in the two CRISPRi LINC00261-depleted cell populations (sgRNA i1, sgRNA i2) compared to empty vector-transduced cells (Table S4). This analysis identified a total of 235 significantly (adjusted *p*-value (*p*-adj.) < 0.05) deregulated genes, with 146 showing increased and 89 showing decreased expression in LINC00261-depleted cells (Figure 5c, upper panel). Moreover, RNA sequencing analysis of three wild-type and three LINC00261 promoter knockout clones (Table S5) revealed a total of 130 significantly (*p*-adj. < 0.05) differentially expressed genes, of which 75 were upregulated and 54 downregulated (Figure 5c, lower panel). Next, we performed gene set enrichment analyses to identify significant deregulated pathways that were individually associated with reduced LINC00261 expression in both knockdown systems. Intriguingly, this unbiased analysis unraveled a significant enrichment of the hallmark gene set “epithelial–mesenchymal transition” in both LINC00261-depleted cell systems (Figure 5d), which is in line with our initial results obtained by in silico analysis in PDAC patient samples (see Figure 2a). These data indicate that the downregulation of LINC00261, especially by TGFβ (see Figure 4f), is of functional relevance. In fact, the decrease of LINC00261 might actively contribute to the establishment of an EMT signature present in the squamous subtype of PDAC. To further narrow down the key downstream targets of LINC00261, we intersected the up- and downregulated genes from both sequencing analyses, which revealed five commonly down- and three commonly upregulated target genes (Figure 5e). Interestingly, three of the eight overlapping genes, namely CDH1, formin 1 (FMN1), and myosin light chain kinase (MYLK), play an important role in EMT or cytoskeletal organization [39,40,41]. Hence, our unbiased gene expression analysis of two distinct LINC00261 knockdown systems revealed a function of LINC00261 in regulating the transcriptional landscape related to EMT.

### 2.5. E-Cadherin Expression is Significantly Downregulated in LINC00261^low^ Cells

**Figure 6:**
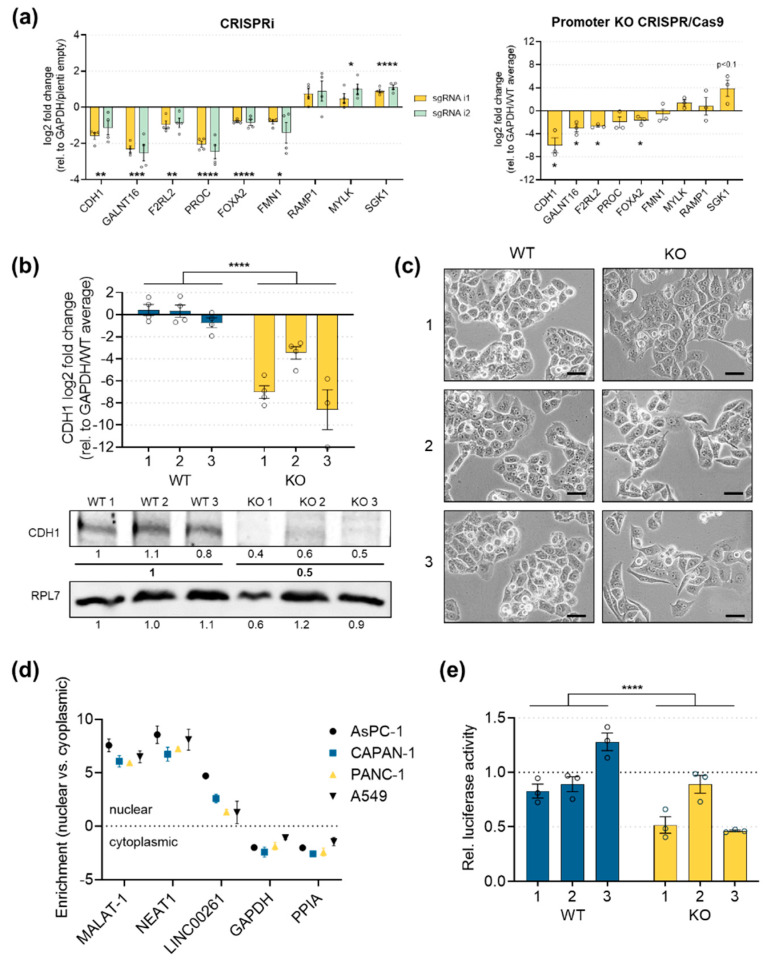
LINC00261 regulated E-cadherin expression. (**a**) The qPCR analysis of the genes commonly deregulated in both CRISPR systems, as shown in Figure 5e, in addition to FOXA2 (* *p* < 0.05, ** *p* < 0.01, *** *p* < 0.001, **** *p* < 0.0001, two-way ANOVA, unpaired *t*-test). (**b**) Expression of E-cadherin mRNA (upper panel) and protein levels (lower panel) in three wild-type and three promoter knockout clones (**** *p* < 0.0001, two-way ANOVA). (**c**) Brightfield images of wild-type and LINC00261 promoter knockout clones (20× objective, scale bar = 50 µm). (**d**) Cellular fractionation in lung cancer and pancreatic cancer cell lines, highlighting nuclear enrichment of LINC00261. (**e**) Relative luciferase activity of the CDH1 gene promoter constructs normalized to the pGL3-vector with a minimal cytomegalovirus (CMV) promotor (pGL3-minCMV). The average of the relative luciferase activity in wild-type clones was set to 1.0 (**** *p* < 0.0001, two-way ANOVA).

In order to validate the RNA sequencing results, we performed qRT-PCR analyses, focusing on the eight commonly up- and downregulated genes in LINC00261^low^ PANC-1 cells. We also analyzed the expression of its genomic neighbor FOXA2, which has been shown to be regulated by LINC00261 in normal and cancerous human and murine cells [42,43,44]. First, we could confirm the significant downregulation of CDH1, FMN1, coagulation factor II receptor-like 2 (F2RL2), polypeptide N-acetylgalactosaminyltransferase 16 (GALNT16), and protein C, inactivator of coagulation factors Va and VIIIa (PROC), as well as the increased expression of receptor activity modifying protein 1 (RAMP1), MYLK, and serum/glucocorticoid regulated kinase 1 (SGK1). Moreover, although RNA sequencing did not show a significant deregulation of FOXA2, subsequent qRT-PCR analysis demonstrated a slight (log2FC = −0.54/−0.63) downregulation of FOXA2 in both cellular systems (Figure 6a). The most interesting downstream target of LINC00261 was CDH1, which was robustly decreased in both systems. The CDH1 gene encodes E-cadherin, an important transmembrane cell adhesion molecule that plays a role in the formation of adherens junctions, thereby contributing to maintaining epithelial cell and tissue structures. Thus, CDH1 is a key epithelial marker that can be regulated by the TGFβ signaling pathway and its associated downstream transcription factors, leading to its downregulation in cells passing through an epithelial-to-mesenchymal transition [45]. Importantly, loss of CDH1 has been shown to be associated with increased invasion or metastasis in several types of carcinoma, and germline mutations have been linked to increased cancer risk [46]. Intriguingly, LINC00261 promoter knockout clones showed a strong and highly significant downregulation of E-cadherin expression on both the RNA (Figure 6b, upper panel) and protein levels in all three selected KO clones (Figure 6b, lower panel). Moreover, the downregulation of E-cadherin in LINC00261-depleted PANC-1 cells induced an EMT-like phenotype with a spindle cell morphology (Figure 6c). These findings support the idea of LINC00261 being involved in maintaining a pro-epithelial cell identity, whereas loss of LINC00261 induces transcriptional and morphological changes, potentially via regulating CDH1 expression. To gain further insights into the potential mechanism of LINC00261-mediated downregulation of E-cadherin, we performed nuclear and cytoplasmic fractionation experiments in lung and pancreatic cancer cell lines to obtain information about the subcellular localization of LINC00261. To verify the purity of the respective fractions, we monitored the enrichment of metastasis associated lung adenocarcinoma transcript 1 (MALAT1) and nuclear enriched abundant transcript 1 (NEAT1), two well-known nuclear lncRNAs [47], as well as the cytoplasmic mRNAs of glyceraldehyde-3-phosphate dehydrogenase (GAPDH) and peptidylprolyl isomerase A (PPIA). All four controls showed expected nuclear or cytoplasmic enrichment, respectively (Figure 6d). More importantly, in all cell lines analyzed, LINC00261 showed a predominantly nuclear enrichment, suggesting a putative function in transcriptional or epigenetic regulation of gene expression. To investigate this further and directly test the idea of a potential transcriptional regulation of CDH1 by LINC00261, we performed luciferase reporter assays. Here, we cloned the promoter region of CDH1 in front of the luciferase gene and transfected this construct into PANC-1 wild-type or LINC00261-depleted cells and measured luciferase activity 48 h later. Intriguingly, the reduced expression level of LINC00261 in KO clones resulted in significantly lower CDH1 promoter activity as compared to WT clones (Figure 6e). Taken together, these results indicate that LINC00261 might be involved in the regulation of CDH1 transcription, thereby controlling the epithelial identity of pancreatic cancer cells.

### 2.6. LINC00261 Downregulation Leads to Increased Cell Migration and Invasion

**Figure 7:**
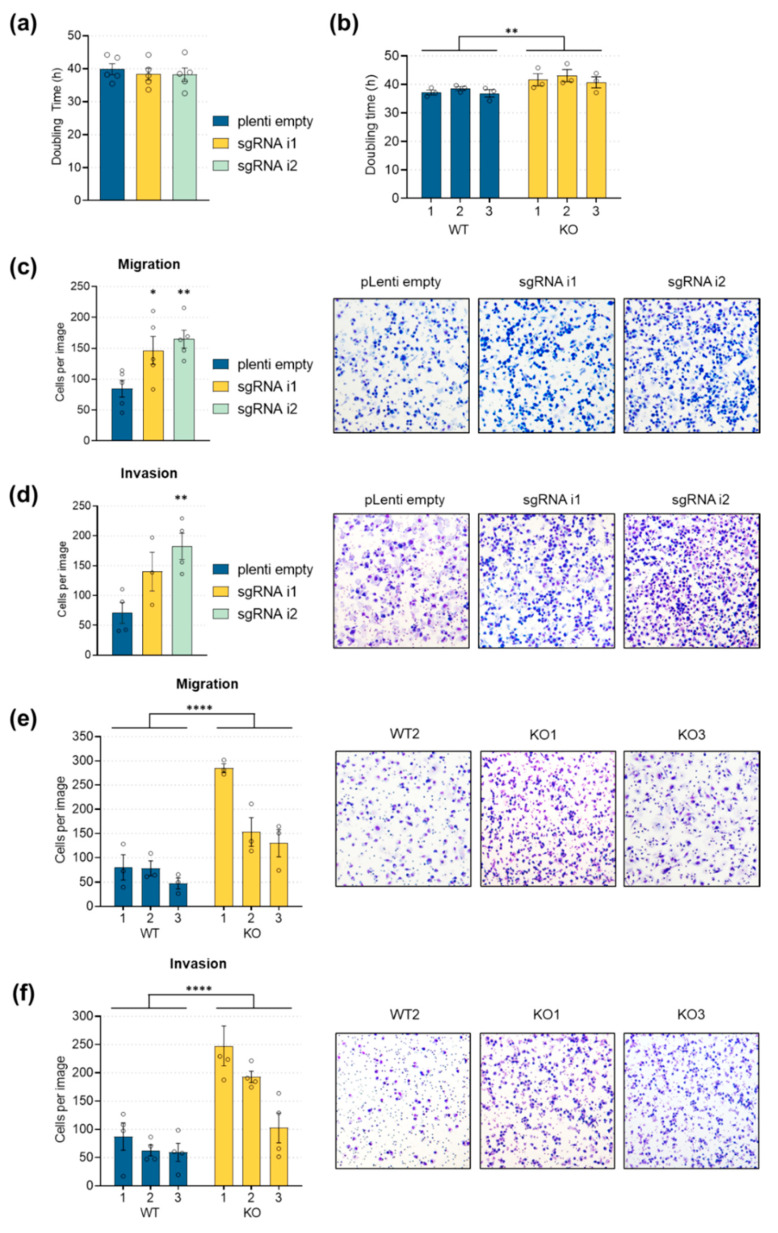
Enhanced cell migration and invasion in LINC00261^low^ cells. (**a**,**b**) Cell doubling time in PANC-1 cells after CRISPRi-mediated LINC00261 downregulation and in three wild-type and three LINC00261 promoter knockout cell clones (** *p* < 0.01, one-way (**a**) and two-way ANOVA (**b**)). (**c**,**d**) Transwell migration (**c**) and invasion (**d**) assays in PANC-1 cells after CRISPRi-mediated LINC00261 downregulation (* *p* < 0.05, ** *p* < 0.01, one-way ANOVA). (**e**,**f**) Transwell migration (**e**) and invasion (**f**) assays in PANC-1 cells after CRISPR/Cas9-mediated knockout of LINC00261 promoter. Quantification of migrated and invaded cells (**c**–**f**) from five random fields of three wild-type and three knockout clones after eosin Y and methylene blue staining using light microscopy (**** *p* < 0.0001, two-way ANOVA).

Downregulation of E-cadherin is a hallmark of EMT and leads to changes in cell polarity, which is crucial for increased cell motility, and therefore directional migration, invasion, and metastasis [45]. Since LINC00261 expression was tightly connected to E-cadherin expression, we hypothesized that LINC00261 might contribute to pancreatic cancer cell properties, especially cancer cell motility. Hence, we characterized the proliferation, migration, and invasion of LINC00261-depleted cells by performing proliferation assays, as well as transwell migration and invasion assays using defined extracellular matrices. Interestingly, the proliferative capacity of the cells was not altered when LINC00261 was knocked down using the CRISPRi approach (Figure 7a). Moreover, LINC00261 promoter knockout clones only showed a slightly extended cell doubling time (Figure 7b). In contrast to these negligible and inconsistent effects on cell proliferation, the influence of LINC00261 expression on cell migration and invasion was striking. In detail, the CRISPRi-mediated reduction of LINC00261 resulted in a ~2-fold increase of cell migration (Figure 7c), as well as a ~2.5-fold increase of invasiveness (Figure 7d).

These findings were confirmed using individual LINC00261 promoter knockout clones, which showed a ~2–4-fold increase in cell migration rate (Figure 7e) and up to a ~4-fold increase of invasion capacity (Figure 7f) compared to wild-type clones. These results are in line with the herein described expression pattern of LINC00261 in PDAC samples and after TGFβ-1 stimulation, as well as the observed transcriptional changes after LINC00261 depletion, especially the downregulation of CDH1. Hence, in pancreatic cancers, the reduction of LINC00261 may trigger the loss of E-cadherin-dependent cell–cell contacts, thereby enhancing the invasive capabilities of pancreatic cancer cells, resulting in a more aggressive subtype of PDAC and a poor survival of patients whose tumors present a low LINC00261 expression.

## 3. Discussion

Recent studies revealed a broad spectrum of lncRNA functions in cancer, including their roles in tumor initiation and progression [13]. However, only a few studies have analyzed the role of lncRNAs in PDAC. Comprehensive and systemic analysis of differential lncRNA expression in pancreatic cancer identified lncRNAs that might be used as disease biomarkers and biomarkers for patient survival [48,49,50,51,52,53]. Overall, a diverse set of lncRNAs in pancreatic cancer has been identified; however, the functions of these biomarkers are largely unknown. Recently, RNA sequencing analyses of large cohorts of PDAC samples brought additional insights into pancreas carcinogenesis. In these studies, different molecular subtypes of prognostic and biological relevance were identified [3,5], and differential expression of lncRNAs was associated with these subtypes [15]. Leveraging the potential of these large datasets, we comprehensively analyzed the cohort of Bailey et al. [3] and applied the NMF algorithm [19] to identify the four previously reported disease subtypes of PDAC. Importantly, PDAC patients assigned to the squamous subtype show the worst overall survival due to a highly aggressive disease histology, which is associated with gene expression changes related to EMT. The EMT process and expression of EMT transcription factors have been linked to cancer progression, as well as therapy resistance [54]. In pancreatic cancer, EMT is associated with tumor cell budding, which is a strong predictor of advanced tumor stage, lymphatic invasion, mortality, and recurrence [55,56]. Moreover, EMT status of patient-derived tumor specimens determined by the expression of epithelial and mesenchymal markers and EMT-associated transcription factors predict the prognosis of pancreatic cancer [57,58]. Interestingly, TGFBR1/2 inhibitors were developed as novel therapeutic strategies to prevent metastasis [59,60] and were shown to inhibit TGFβ-mediated LINC00261 downregulation in our study (see Figure 4g). In contrast, another study showed that EMT might be dispensable for metastasis but important for chemoresistance in pancreatic cancer [61]. Similar conclusions were drawn for breast cancer [62]. However, both studies are highly debated [63,64]; a recent study from the Brabletz lab could show that genetic ablation of the EMT-activator ZEB1 in a murine pancreatic cancer model blocks invasion and metastasis, highlighting the non-redundant roles of EMT transcription factors [65,66]. Since lncRNAs have been shown to affect gene expression on multiple levels, we hypothesized that these transcripts could actively contribute to the disease biology of the aggressive squamous subtype of PDAC by modulating the expression of epithelial and mesenchymal genes, thereby supporting the migratory and invasive phenotypes of cancer cells. Hence, we analyzed the lncRNA expression landscape across PDAC subtypes in order to identify lncRNAs specifically associated with the squamous subtype. This led to the identification of LINC00261, whose expression was variable across PDAC subtypes and correlated with stage and grade, as well as favorable patient survival (see Figure 1). More specifically, we found a significant downregulation of LINC00261 expression in the squamous subtype of the Bailey dataset [3] and in the basal-like subtype of the TCGA dataset, as defined by Moffitt et al. [5], including only those samples with a high tumor cell content, as described by the Cancer Genome Atlas Research Network [15]. Intriguingly, a positive correlation of LINC00261 expression was found for genes that are important for determining the fate of endodermal cells, such as PDX1 or GATA6, whose expression is low in squamous tumors, implicating LINC00261 as a functional lncRNA important for endoderm differentiation. Indeed, this was shown recently in human embryonic stem cells [42]. On the other hand, a strong negative correlation exists between LINC00261 and YAP1, which was identified as an oncogenic driver specifically in squamous tumors [20]. Although these correlation analyses do not prove a causal role of LINC00261 in driving the squamous subtype, it is very likely that LINC00261 is one of several important factors that contribute to the establishment of a gene expression program that is characteristic for the squamous subtype of pancreatic cancer. Compared to normal pancreatic tissue, LINC00261 expression was significantly lower in pancreatic cancer, which is in accordance with earlier reports in other tumor types showing deregulated LINC00261 expression in cancer versus normal tissue [17,18,35]. Moreover, higher LINC00261 expression was found in low-grade and early–stage PDAC samples. Altogether, our analyses in pancreas carcinoma, along with additional published reports in other cancer entities, strongly suggest that LINC00261 might function as a tumor-suppressive lncRNA. However, a study on cholangiocarcinoma found that LINC00261 might also have pro-tumorigenic functions [67]. Further, in silico analysis in LINC00261^high^ versus LINC00261^low^ tumors confirmed the previously demonstrated association with EMT in a very comprehensive way. We validated this association using correlation analyses, which revealed an inverse correlation of mesenchymal genes with LINC00261 expression in all studied datasets. An important EMT inducer is TGFβ, which has a dual action in cancer, acting as a tumor suppressor and oncogene, due to its effector functions on tumor cells and on cells of the tumor microenvironment [68]. High activity of the TGFβ pathway is associated with poor prognosis in pancreatic cancer, as well as in other cancer types [3,5,15,69]. Activation of the TGFβ signaling pathway, especially in later stages of tumor progression, can result in the acquisition of mesenchymal features in cancer cells [54], increased resistance to chemotherapeutic agents [61], and can support immune evasion [70]. Here, we could show that the stimulation of lung and pancreatic cancer cells induced a fast and strong downregulation of LINC00261 expression in cells that pass through an EMT (TGFβ-responsive), but not in non-responsive cells that fail to acquire a mesenchymal morphology associated with a cadherin switch. Thus, LINC00261 can be added to the list of bona fide TGFβ downstream targets. However, the functional relevance of LINC00261 in the context of TGFβ signaling and EMT was still unknown at this point. Hence, to address the question of whether the downregulation of LINC00261 might contribute to implement an EMT-related gene expression network, we leveraged two complimentary CRISPR strategies to reduce LINC00261 levels in PANC-1 cells. Surprisingly, we found a significant enrichment of genes related to EMT after LINC00261 depletion using CRISPR interference or CRISPR/Cas9-mediated LINC00261 promoter deletion. Intriguingly, the important epithelial marker E-cadherin was robustly downregulated in both LINC00261-depleted cell systems. These findings are supported by a very recent publication by Chen et al. [71]. Here, the authors found that overexpression of LINC00261 in PANC-1 and MIA-PaCa2 cells strongly increased E-cadherin expression, while its shRNA-mediated depletion decreased E-cadherin. Mechanistically, Chen and colleagues suggested that LINC00261 might function as a competing endogenous RNA (ceRNA) by sponging miR-552-5p, which in turn increased FOXO3 levels to regulate the Wnt pathway [72]. Furthermore, they showed that overexpression of LINC00261 reduced β-catenin and TCF4 expression and simultaneously increased E-cadherin, which is a TCF4 downstream target. In addition, other studies suggested that LINC00261 might act as a sponge for several miRNAs, as it was found in several comprehensive analyses of ceRNA networks in different types of cancer [73,74,75]. However, stringent experimental evidence that would clearly support a ceRNA function of LINC00261 is still lacking. Importantly, while the study of Chen and colleagues largely complements and supports major findings of our study, we did not identify FOXO3, β-catenin, or TCF4 as commonly deregulated genes, and TCF4 expression was only significantly upregulated (log2FC = 1.76) in LINC00261 promoter knockout clones rather than wild-type clones. Moreover, our own cell fractionation analysis in a panel of lung and pancreas cell lines revealed a predominant nuclear localization of LINC00261, which is supported by other studies in mouse hepatocytes [43], esophageal cancer cells [76], and lung epithelial cells [44]. This localization pattern might suggest a role for LINC00261 in the control of target gene transcription, e.g., through the recruitment of undefined transcription factors or by the regulation of higher order chromatin folding. Both possibilities will be tested in the future. However, nuclear enrichment of LINC00261 is not as prominent as it is for well-known nuclear lncRNAs such as MALAT1 or NEAT1, which were included as positive controls in our experiments [47]. Thus, LINC00261 might also shuttle between the cytoplasm and the nucleus, and could have different molecular functions depending on its subcellular localization. Moreover, the localization or function of LINC00261 might also differ between normal and cancer cells or even between different cancer types, which also warrants further investigation to fully understand the regulation and biological role of LINC00261 in cancer. Here, we could confirm its direct regulation by TGFβ. In addition, we could show that LINC00261 actively contributed to an EMT gene expression signature and its depletion caused morphological changes, potentially by regulating CDH1 expression. Importantly, it has been shown that a decrease of CDH1 expression can solely be responsible for pancreatic cancer metastasis [77]. Additionally, proteomic analyses revealed that low CDH1 correlated with poor disease outcome [78]. The reduced expression of LINC00261 in the squamous subtype of PDAC, which is characterized by the loss of E-cadherin expression and the induction of a mesenchymal cell identity, therefore might be causally linked to disease progression rather than only being a bystander effect. This idea is supported by our own in vitro experiments, which revealed a strong induction of cell migration and invasion after LINC00261 downregulation. However, the detailed mechanism of regulation of CDH1 expression by LINC00261 and potentially involved transcription factors still needs to be unraveled. Known negative regulators of CDH1 gene expression are SNAI1, SNAI2, TWIST, and ZEB1 [79,80,81,82,83,84], whereas AML1, p300, HNF1α, and FOXA2 seem to positively regulate CDH1 expression [85]. In a previous study it was shown that LINC00261 could bind to SNAI2 and promote its degradation in gastric cancer [35]. However, similar to TWIST, the expression of SNAI2 is rather low in PANC-1 cells, meaning this EMT transcription factor might not play an important regulatory role in this cell system. Future work will include analysis of interactions with other EMT-TFs, including ZEB1 and SNAI1. Several studies have highlighted these TFs as main inducers of EMT conversion in different types of cancer, especially in response to stimuli such as TGFβ and NF-κB [86,87,88]. Furthermore, CDH1 expression seems to be highly dependent on the methylation status of its promotor, and an epigenetic function of LINC00261 should also be considered in future investigations [89,90]. Additionally, we observed a regulatory circuit between FOXA2 and LINC00261 in both directions, suggesting that LINC00261 could regulate CDH1 expression through FOXA2, since CDH1 has been shown to be regulated by FOXA2 [31,85,91]. Control of FOXA2 expression by LINC00261 has also been observed in lung cancer cells and mouse hepatocytes [34,43,44]. FOXA2 itself is suggested to be important for pancreas development [33] and potential tumor suppression [30,31,32]; however, controversy exists as to whether FOXA2 could also act as an oncogene in some cancer types [92,93]. Our results indicate that LINC00261 expression is transcriptionally regulated by FOXA2 through direct binding to the LINC00261 promoter, as demonstrated with ChIP and luciferase analyses. These results are supported by studies of lung cancer that have indicated a tight interconnection between these two genes [17,34,94]. However, to further determine the regulatory consequences and to investigate the biological and therapeutic relevance of LINC00261 for PDAC growth, invasion, or metastasis, one should consider additional model systems, such as organoids [95,96,97] or mouse models [98,99].

In summary, our study established LINC00261 as a tumor-suppressive lncRNA in PDAC. The downregulation of LINC00261, as it occurs in the progression of PDAC, may contribute to the EMT of pancreatic cancer cells, at least partly due to its direct effect on E-cadherin. These results may help to understand the molecular mechanisms underlying the development and metastasis of PDAC and highlight LINC00261 as a novel prognostic and diagnostic biomarker. However, additional studies are needed to fully understand the molecular functions of LINC00261 and to develop therapeutic strategies to restore its expression in tumors.

## 4. Materials and Methods

### 4.1. Bioinformatics Analysis of LINC00261 Expression in PDAC samples

For clustering of the ICGC PDAC samples according to their RNA expression, we used the normalized expression values provided by the supplementary table in the paper by Bailey et al. [3]. First, we selected the 2000 genes showing the highest variation in their expression values, using the coefficient of variation as a measure of variability. Since the normalized expression data contained negative values, we added the overall minimal value of these 2000 genes as a constant to all expression values in order to obtain only positive expression values. Subsequently, we applied non-negative matrix factorization using the R-package NMF [19], using Brunet’s algorithm, rank = 4, and 500 iterations. Each sample was then assigned to the cluster with the highest corresponding likelihood. Differential expression analysis between pancreatic ductal adenocarcinomas and normal pancreatic tissue samples was performed as follows. We obtained gene-level RNA-seq read counts of TCGA primary tumor PDAC samples and GTEx V7 normal pancreas tissue via the GDC data portal (portal.gdc.cancer.gov) and the GTEx portal (gtexportal.org), respectively. By combining these data, we obtained read count information of 53045 genes for 177 primary tumor samples and 248 normal pancreas tissue samples. Differential gene expression was assessed using R/edgeR [100] by applying Trimmed Mean of M-values (TMM) normalization. Counts per Million (CPM) transformation was utilized to obtain normalized expression values. Kaplan–Meier and gene expression correlation analyses of the Bailey PDAC dataset were determined using the R2: Genomics Analysis and Visualization Platform (http://r2.amc.nl).

### 4.2. RNA Extraction from PDAC and Normal Pancreas Tissue Samples

Formalin-fixed, paraffin-embedded (FFPE) blocks of normal and cancerous human pancreatic tissues were obtained from the Institute of Pathology, Martin Luther University Halle-Wittenberg after approval by the Ethics Committee of the Medical Faculty, Martin Luther University Halle-Wittenberg (no. 2015-016, no. 2017-81). RNA was extracted from 34 normal pancreas and 42 PDAC tissue blocks with tumor cell content >65%. Total RNA was extracted from three 10 µm paraffin sections using the RNeasy FFPE Kit (Qiagen, Hilden, Germany) according to the manufacturer’s instructions. Then, 1 µg of total RNA was transcribed into cDNA for subsequent quantitative real-time PCR (qRT-PCR). Patient characteristics are provided in Table S2.

### 4.3. Cell Culture and siRNA Transfection

The human pancreatic cancer cell lines (AsPc-1, Capan-1, PANC-1), the human lung cancer cell line A549, and the human embryonic kidney (HEK)293T cells were purchased from American Type Culture Collection (ATCC, Manassas, VA, USA). PANC-1, Capan-1, A549, and HEK293T cells were grown in Dulbecco’s modified Eagle’s medium (DMEM, Life Technologies, Carlsbad, CA, USA) supplemented with 10% fetal bovine serum (FBS, Thermo Fisher Scientific, Waltham, MA, USA) and 1% penicillin/streptomycin (Thermo Fisher Scientific). The pancreatic cancer cell lines AsPC-1 was maintained in RPMI-1640 medium (Thermo Fisher Scientific) supplemented with 10% fetal bovine serum and 1% penicillin/streptomycin. Cells were maintained at 37 °C in a humidified incubator infused with 20% O_2_ and 5% CO_2_. All pancreatic cancer cell lines harbored mutations in KRAS and p53 resembling human PDAC [101], and showed metastatic potential in in vivo settings [102]. For TGFβ-1 treatment, 1.0–1.5 × 10^6^ cells of each cell line that had been plated on a 10 cm plate the previous day were starved for 24 h using medium supplemented with 0.5% FBS and then treated with 10 ng/µL TGFβ-1 (PeproTech, Rocky Hill, CT, USA, diluted in 0.5% FBS in DMEM or RPMI-1640). Cell lines were harvested after 24, 48, and 72 h of TGFβ-1 treatment for protein and RNA isolation. For the treatment with the TGF-β type 1 receptor (TGFBR1) inhibitor RepSox (Selleckchem, Houston, TX, USA, 200 nM f.c.), 1.5 × 10^5^ A549 and 2 × 10^5^ PANC-1 cells were seeded on a 6-well plate, starved in DMEM containing 0.5% FBS for 24 h, treated in the same medium, and harvested after 24, 48, and 72 h for RNA isolation. The siRNA transfection of PANC-1 cells with two independent FOXA2 siRNAs (Table S3) was performed with 20 nM final concentration of siRNA by using Lipofectamine RNAiMax (Thermo Fisher Scientific) according to the manufacturer’s instructions. Cells were harvested for RNA isolation72 h after transfection.

### 4.4. Genomic DNA and Total RNA Extraction Followed by qRT-PCR

Genomic DNA was extracted from cell pellets (~3 × 10^6^ cells) by using the ReliaPrep™ gDNA Tissue Miniprep System (Promega, Madison, WI, USA)) according to manufacturer’s instructions. Total RNA was extracted from cell lines and tissues by using Trizol reagent as described previously [103]. Then, 1 μg RNA was reversely transcribed to cDNA using Moloney Murine Leukemia Virus (M-MLV) Reverse Transcriptase and the provided 5× reaction buffer (Promega) according to manufacturer’s instructions. Subsequently, qRT-PCR was carried out in triplicate in a 384-well plate with the LightCycler 480 system (Roche Life Science, Indianapolis, IN, USA) using 6.25 ng cDNA, 0.7 µM forward and reverse primers, and primaQUANT qPCR SYBR Green Master Mix (Steinbrenner Laborsysteme, Wiesenbach, Germany). Primers used for qRT-PCR are listed in Table S3. GAPDH amplification was used as reference for qRT-PCR. Relative expression values were calculated according to the 2−ΔΔCt method [104].

### 4.5. Protein Extraction and Western Blot Analysis

Cells were washed twice with PBS (Thermo Fisher Scientific) and lysed in RIPA lysis buffer (50 mM Tris-HCl, pH 8.0, 150 mM NaCl, 1% IGEPAL CA-630, 0.5% Sodium deoxycholate, 0.1% SDS) supplemented with cOmplete EDTA-free EASYpack protease inhibitor (Roche Life Science, Indianapolis, IN, USA). The extracted proteins were separated by 10% sodium dodecyl sulfate-polyacrylamide gel electrophoresis (SDS-PAGE) and transferred onto a nitrocellulose membrane (GE Healthcare, Piscataway, NJ, USA). Prior to antibody incubation, the membranes were blocked with 5% skimmed milk in Tris-based saline with 0.1% Tween 20. Diluted primary antibodies in blocking solution were added overnight at 4 °C. The following primary antibodies were purchased from Cell Signaling Technology (Danvers, MA, USA): E-cadherin (#3195, 1:1000), CDH2 (#13116, 1:1000), VIM (#5741, 1:1000), SMAD2 (#5339, 1:1000), p-SMAD2 (#18338, 1:1000), SMAD3 (#9523, 1:1000), p-SMAD3 (#9520, 1:1000), FOXA2 (#8186, 1:1000). Additional antibodies used were GAPDH (Sigma-Aldrich, St. Louis, MO, USA, #G8795, 1:5000) and RPL7 (Bethyl Laboratories, Montgomery, TX, USA, #A400-741A, 1:1000). Secondary antibodies (IRDye^®^ 800CW/680CW anti-mouse/rabbit, LI-COR Biosciences, Lincoln, NE, USA) were added for 2 h at room temperature. Antibody signals were visualized using the Odyssey infrared scanner (LI-COR Biosciences). Uncropped blots with molecular weight markers and densitometry readings are provided in Supplementary Figure S5.

### 4.6. Subcellular Fractionation

Subcellular fractionation was performed as described earlier [105]. In brief, A549, AsPC-1, CAPAN-1, and PANC-1 cells were scraped off the plate in their respective growth medium and pelleted at 500× *g* for 5 min at 4 °C. Pellets were washed once with PBS, centrifuged at 800× *g* for 5 min at 4 °C, resuspended in 1 mL of RSB (10 mM Tris, pH 7.4, 10 mM NaCl, 3 mM MgCl_2_), incubated for 3 min on ice, and centrifuged at 1000× *g* for 5 min at 4 °C. One-fifth of the total cells was removed for isolation of total RNA. The cell pellet was resuspended with four times its volume of RSBG40 (10 mM Tris, pH 7.4, 10 mM NaCl, 3 mM MgCl_2_, 10% glycerol, 0.5% Nonidet P-40, 0.5 mM dithiothreitol) supplemented with 40 U/mL Ribolock (Thermo Fisher Scientific) and 5 mM ribonucleoside vanadyl complex (New England Biolabs, Ipswich, MA, USA). After incubation for 3 min on ice and centrifuging at 4500× *g* for 3 min at 4 °C, the supernatant was saved as the cytoplasmic fraction, and the nuclear pellet was resuspended in RSBG40 containing one-tenth volume of detergent (3.3% (wt/wt) sodium deoxycholate and 6.6% (vol/vol) Tween-20) and incubated for 5 min on ice. Nuclei were pelleted again at 4500× *g* for 3 min at 4 °C, washed with RSBG40, and collected at 9300× *g* for 5 min. RNA from nuclear and cytoplasmic fraction was isolated using Trizol.

### 4.7. Chromatin Immunoprecipitation

ChIP assays were performed using the SimpleChIP™ Enzymatic Chromatin IP Kit (#9003, Cell Signaling Technology) according to manufacturer’s instructions. Briefly, untreated or TGFβ-treated PANC-1 cells were fixed with 1% formaldehyde to crosslink DNA and proteins, chromatin was sheared using a UP200S Lab Homogenizer (Hielscher Ultrasonics, Teltow, Germany, 3 cycles of sonication: 20” each, 30” rest; amplitude 30%), and 10 µg of the chromatin fraction as incubated with 0.5 µg of antibodies specific for FOXA2 (#8186, Cell Signaling Technology), histone H3 (#4620, Cell Signaling Technology, positive control) and IgG (#2729, Cell Signaling Technology, negative control). The complex was precipitated by Protein G magnetic beads (30 μL). The protein–DNA cross-link was reversed, the DNA was purified, and the enrichment of DNA sequences was detected using qPCR. The primers used in this study are listed in Table S3, while the genomic locations of the analyzed regions are indicated in Figure 3e.

### 4.8. Cloning

The coding sequence of FOXA2 was amplified by PCR from the EF1a_FOXA2_P2A_Hygro_Barcode vector (Addgene #120439, gift from Prashant Mali) and cloned into pCDH-CMV-MCS-EF1-Puro (System Biosciences, Palo Alto, CA, USA) using NheI/BamHI restriction sites. Sequence of primers used are provided in Table S3. Lentivirus-carrying pCDH-CMV-FOXA2-EF1-Puro plasmid was produced in HEK293T cells, and transduced cells were selected by adding 2 µg/mL Puromycin (Thermo Fisher Scientific) to the culture medium. For promoter analysis, the E-cadherin promoter region from −770 to +92 and the LINC00261 putative promoter region from −1000 to +100 were amplified using primers with restriction enzyme sites of NheI/XhoI (E-cadherin promoter) or XhoI/HindIII (LINC00261 promoter, Table S3) from genomic DNA of PANC-1 cells. Amplified PCR products were inserted into the upstream region of the firefly luciferase gene of the pGL3-Basic vector (Promega). Similarly, an oligo containing the sequence of a minimal CMV promoter was inserted into the pGL3-Basic vector and used as a control plasmid for all luciferase experiments. Single-guide RNA (sgRNA) targeting the LINC00261 gene with CRISPRi and for cutting out the LINC00261 promoter was designed using the Broad Institute CRISPR design tool (https://portals.broadinstitute.org/gpp/public/analysis-tools/sgrna-design). For CRISPRi, two independent sgRNAs were selected and cloned into the lentiGuide-Puro plasmid (Addgene #52963, gift from Feng Zhang). For this purpose, oligonucleotides containing the sgRNA-expressing sequence and BsmBI sticky ends were synthesized (Eurofins Genomics, Ebersberg, Germany), annealed, phosphorylated, and ligated with the vector. Mach-1 competent cells were used for transformation. The sgRNA sequences are provided in Table S3. To remove the putative promoter of LINC00261, the two sgRNAs were cloned into the pX330-Cas9-P2A-mCherry vector (Addgene #98750, gifted by Jinsong Li) and the pL-CRISPR.EFS.GFP (Addgene #57818, gift from Benjamin Ebert) in the same way.

### 4.9. LINC00261 Downregulation by CRISPR Interference

Lentivirus was produced in HEK293T cells (4 × 10^6^ in 10 cm plate). Briefly, the Lenti-dCas9-KRAB-blast plasmid (10 µg, Addgene #89567, gift from Gary Hon) or the sgRNA coding plasmids (10 µg) were transfected together with lentiviral packaging plasmids psPAX2 (5 µg, Addgene #12259, gift from Didier Trono) and pMD2.G (2.5 µg, Addgene #12259, gift from Didier Trono) using TurboFect reagent (Thermo Fisher Scientific) according to the manufacturer’s instructions. The virus was harvested 72 h after transfection. First, transduction of the lenti-dCas9-KRAB-blast plasmid in 3 × 10^5^ PANC-1 cells (6-well plate) was performed. Two days later, cells were treated with 10 µg/mL Blastidicin (Santa Cruz, Dallas, TX, USA) in order to select for cells that were transduced with the plasmid. Subsequently, the lenti-dCas9-KRAB-blast PANC-1 cells were transduced with the sgRNA coding plasmids (lentiGuide-Puro as control, CRISPRi sgRNAs targeting LINC00261 named sgRNA i1 and i2) for 48 h and selected by treating them with 2 µg/mL Puromycin (Thermo Fisher Scientific).

### 4.10. LINC00261 Promoter Knockout Using CRISPR/Cas9

Next, 3 × 10^5^ cells/well in 3 mL antibiotic-free standard growth medium were seeded in 6-well plates 24 h prior to transfection. For transfection, 4 µg of pL-CRISPR.EFS.GFP-sgLINC00261 and pX330-Cas9-P2A-mCherry-sgLINC00261 were mixed with TurboFect reagent (Thermo Fisher Scientific) according to the manufacturer’s instructions. Then, 72 h post-transfection, mCherry/GFP double-positive single cells were sorted into 96-wells using FACSMelody (BD Biosciences, Franklin Lakes, NJ, USA). Cell clones were expanded, and gDNA and RNA were isolated to check for possible knockout.

### 4.11. RNA-Seq and Data Analysis

Total RNA was isolated using Trizol. RNA integrity and quantitation were assessed using the RNA Nano 6000 Assay Kit of the Bioanalyzer 2100 system (Agilent Technologies, Santa Clara, CA, USA). Library preparation and sequencing was performed by Novogene (HK). In detail, 1 µg RNA per sample was used as the input material for the RNA sample preparations. Sequencing libraries were generated using NEBNext^®^ Ultra™ Directional RNA Library Prep Kit (New England Biolabs) following the manufacturer’s recommendations and index codes were added to attribute sequences to each sample. Briefly, mRNA was purified from total RNA using poly-T oligo-attached magnetic beads. Fragmentation was carried out using divalent cations under elevated temperature in NEBNext first-strand synthesis reaction buffer (5×). First-strand cDNA was synthesized using a random hexamer primer and M-MuLV reverse transcriptase (RNaseH-). Second-strand cDNA synthesis was subsequently performed using DNA Polymerase I and RNase H. In the reaction buffer, dNTPs with dTTP were replaced by dUTP. The remaining overhangs were converted into blunt ends via exonuclease and polymerase activities. After adenylation of 3’ ends of DNA fragments, the NEBNext adaptor with a hairpin loop structure was ligated to prepare it for hybridization. In order to select cDNA fragments measuring 250–300 bp in length, the library fragments were purified with the AMPure XP system (Beckman Coulter, Beverly, NJ, USA). Then, 3 μL Uracil-Specific Excision Reagent (USER®) enzyme (New England Biolabs) was used with size-selected, adaptor-ligated cDNA at 37 °C for 15 min, followed by 5 min at 95 °C before PCR. Then, PCR was performed with Phusion HighFidelity DNA polymerase, Universal PCR primers, and Index(X) primer. Finally, the products were purified (AMPure XP system, Beckman Coulter, Brea, CA, USA) and the library quality was assessed using the Agilent Bioanalyzer 2100 system (Agilent Technologies). The clustering of the index-coded samples was performed on a cBot Cluster Generation System using the cBot-HS PE Cluster Kit (Illumina, San Diego, CA, USA) according to the manufacturer’s instructions. After cluster generation, the library preparations were sequenced on a Novaseq 6000 platform and paired-end reads were generated. Raw data (raw reads) in FASTQ format were firstly processed through in-house scripts. In this step, clean data (clean reads) were obtained by removing reads containing adapter and poly-N sequences and reads of low quality from raw data. At the same time, the Q20, Q30, and GC contents of the clean data were calculated. All the downstream analyses were based on the high-quality clean data. The analysis of the RNA-seq datasets was performed by using the Galaxy web platform (https://usegalaxy.eu/). At first, reads with a minimum of 20 bp were aligned to human genome build GRCh38/hg38 using Spliced Transcripts Alignment to a Reference (STAR) [106]. Subsequently, we used the featureCounts tool [107] to count reads according to GRCh38.87 human gene annotation. Next, we calculated the differently expressed genes using the DESeq2 tool [108]. The list of differently expressed genes (Tables S4 and S5) was used for gene set enrichment analysis (GSEA, https://www.gsea-msigdb.org/gsea/index.jsp) to identify specifically enriched hallmark gene sets. Overlaps of genes are shown with Venn diagrams, which were generated using an online available Venn tool (http://bioinformatics.psb.ugent.be/webtools/Venn).

### 4.12. Luciferase Assay

The 2 × 10^5^ PANC-1 cells were seeded into each well of a 6-well plate. Then, 24 h later the cells were transfected with 500 ng of the pGL3-CDH1 and pGL3-LINC00261 promoter or the control pGL3-minCMV promoter construct using TurboFect reagent (Thermo Fisher Scientific). For normalization purposes, 10 ng of pRL-SV40 Renilla expression construct (Promega) was used for each transfection. Cell extracts were prepared and the luciferase activity was measured 48 h after transfection by using the Dual-Luciferase Reporter System Kit (Promega). Relative luciferase activity was calculated using pGL3-minCMV as control.

### 4.13. 2D Cell Proliferation Assay

To determine 2D cell proliferation, 5 × 10^3^ cells were plated in 96-well plates and incubated 24 h prior to the first confluence measurement using the IncuCyte live cell analysis imaging system (Sartorius, Göttingen, Germany). Measurements were performed every 6 h up to 72 h. The growth curve was determined by the IncuCyte analysis software and the doubling time was calculated from the growth curve.

### 4.14. Cell Migration and Invasion Assays

Transwell migration and invasion assays were performed using transwell inserts with 8 µm^2^ pore size (Corning, Corning, NY, USA). The membranes were coated with 100 µL migration matrix (0.1% gelatin (G9391, Sigma-Aldrich) in 0.02 M acetic acid) or invasion matrix (50 µg/mL collagen IV (C5533, Sigma-Aldrich), 5 µg/mL laminin (L6274, Sigma-Aldrich), 2 mg/mL gelatin) and incubated at room temperature for 2 h on a rotating platform. The remaining liquid was removed and the membrane dried for 1 h under sterile conditions. PANC-1 cells (7.5 × 10^4^) were plated in the upper chamber in 100 µL serum-free medium. The lower chamber was filled with 500 µL of complete medium containing FBS and antibiotics. After 12 h of incubation, non-migrated cells of the upper chamber were removed using a cotton swab and migrated cells on the bottom of the membrane were fixed and stained using the Richard-Allan Scientific Three-Step Stain Set (Thermo Fisher Scientific). The number of migrated or invaded cells was determined by taking five images per transwell chamber using 20× magnification and the ImageJ cell counter (https://fiji.sc/). The results were calculated as the average of the cells per image for all five images.

### 4.15. Statistical Analysis

All data were reported as standard error of the mean (SEM). Results were analyzed using Student’s t-test, Mann–Whitney test, or one- or two-way ANOVA test, as required. Statistical analyses were performed using GraphPad Prism software 8.0 (GraphPad Software, San Diego, CA, USA) and the difference was considered significant when *p* < 0.05 (* *p* < 0.05; ** *p* < 0.01; *** *p* < 0.001; **** *p* < 0.0001). Experiments were repeated at least three times.

## 5. Conclusions

In this study, we identified LINC00261 as a crucial lncRNA downregulated in the squamous subtype of PDAC, which is characterized by the worst prognosis. Consistently, LINC00261 expression was inversely correlated with disease stage, grade, and patient survival. LINC00261 was downregulated by TGFβ and was regulated by its genomic neighbor FOXA2. Furthermore, downregulation of LINC00261 altered the epithelial identity of PDAC cells by decreasing CDH1 levels and inducing an EMT-related transcription program that enhanced cancer cell invasion and migration. Our results establish LINC00261 as a tumor-suppressive lncRNA that might be a promising therapeutic target and a prognostic and diagnostic biomarker in PDAC.

## Supplementary Materials

The following are available online at https://www.mdpi.com/2072-6694/12/5/1227/s1: Figure S1: Scatter plots of LINC00261 gene expression and genes important for endodermal cell-fate determination and downregulated in the squamous subtype of the Bailey dataset (**a**). Genes reported to be upregulated in the squamous subtype (**b**). Pearson correlation analysis was performed to determine statistical significance. PDX1, Pancreatic And Duodenal Homeobox 1; GATA6, GATA Binding Protein 6; MNX1, Motor Neuron And Pancreas Homeobox 1; EGFR, Epidermal Growth Factor Receptor; LOX, Lysyl Oxidase; YAP1, Yes-Associated Protein 1. Figure S2: Analysis of the Bailey PDAC dataset revealed a significant downregulation of FOXA2 expression in the squamous (S) compared to the pancreatic progenitor (P), immunogenic (I), and ADEX (A) subtypes (**** *p* < 0.0001; one-way ANOVA). Figure S3: FOXA2 expression in TGFβ-responsive and non-responsive cells using qRT-PCR (* *p* < 0.05, ** *p* < 0.01, *** *p* < 0.001; unpaired t-test). Figure S4: ChIP followed by qPCR analysis showed reduced binding of FOXA2 to the LINC00261 promoter in all 3 experiments performed; however, the difference did not reach statistical significance (* *p* < 0.05, *** *p* < 0.001; unpaired t-test). Figure S5: Uncropped blots with molecular weight markers and densitometry readings for Figure 3c, Figure 4d, Figure 4e, and Figure 6b. Table S1: Differentially expressed genes in squamous vs. other subtypes associated with overall survival. Table S2: Patient characteristics (FFPE samples). Table S3: Sequences of PCR primers, cloning oligonucleotides, siRNAs, and sgRNAs. Table S4: Differential gene expression between pLenti empty and LINC00261 i1/i2. Table S5: Differential gene expression between WT and LINC00261 promoter KO PANC-1.
