# Supplementary material for: LINC00261 Is Differentially Expressed in Pancreatic Cancer Subtypes and Regulates a Pro-Epithelial Cell Identity
Source: Cancers (Basel). 2020 May 13;12(5):1227. doi: 10.3390/cancers12051227 (PMC7281485; doi:10.3390/cancers12051227)
Supplement: Supplementary file 1 [file cancers-12-01227-s001.zip › Supplementary Material/cancers-772776-suppl figures org_corrected.docx]

Article

LINC00261 is Differentially Expressed in Pancreatic Cancer Subtypes and Regulates a Pro-Epithelial Cell Identity

Agnes Dorn, Markus Glaß, Carolin T. Neu, Beate Heydel, Stefan Hüttelmaier,
Tony Gutschner, and Monika Haemmerle

Supplementary Material


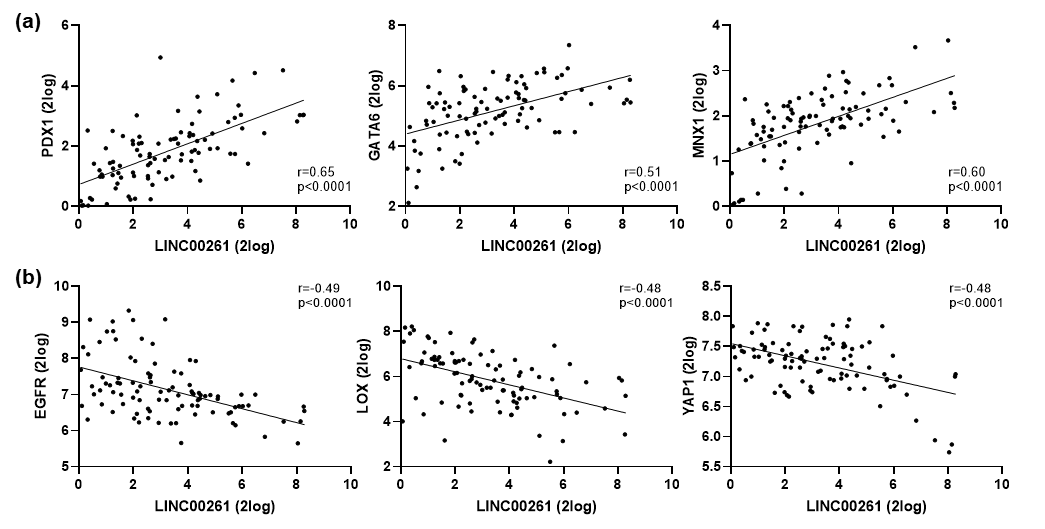


**Figure S1:** Scatter plots of LINC00261 gene expression and genes important for endodermal cell-fate determination and downregulated in squamous subtype of the Bailey dataset (**a**) and genes reported to be upregulated in the squamous subtype (**b**)**.** Pearson correlation analysis was performed to determine statistical significance. PDX1, Pancreatic And Duodenal Homeobox 1; GATA6, GATA Binding Protein 6; MNX1, Motor Neuron And Pancreas Homeobox 1; EGFR, Epidermal Growth Factor Receptor; LOX, Lysyl Oxidase; YAP1, Yes-Associated Protein 1.


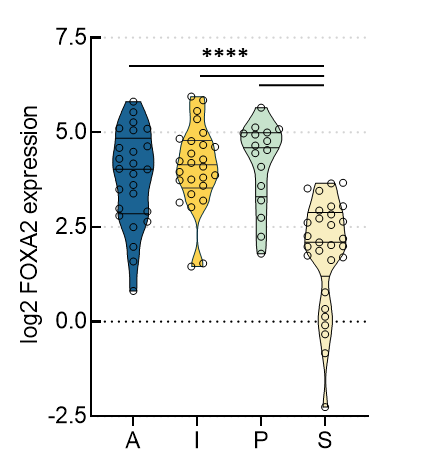


**Figure S2:** Analysis of the Bailey PDAC dataset revealed a significant downregulation of FOXA2 expression in the squamous (S) compared to the pancreatic progenitor (P), immunogenic (I) and ADEX (A) subtype (**** *p* < 0.0001; one-way ANOVA).

**
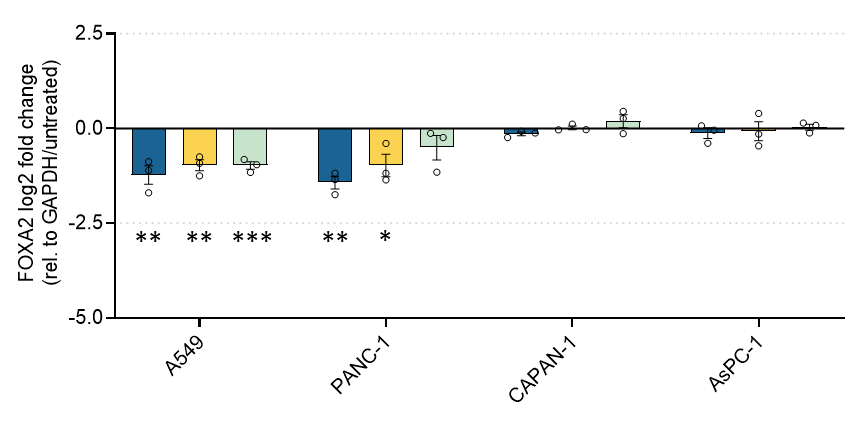
**

**Figure S3.** FOXA2 expression in TGFβ-responsive and non-responsive cells using qRT-PCR (* *p* < 0.05, ** *p* < 0.01, *** *p* < 0.001; unpaired t-test).


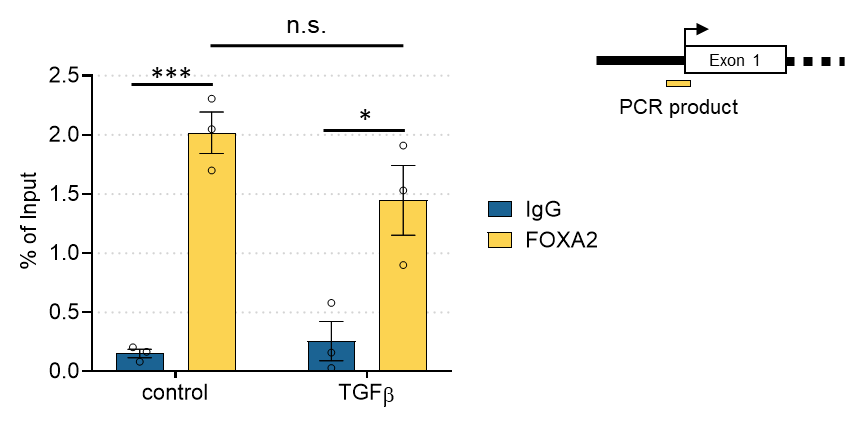


**Figure S4.** ChIP followed by qPCR analysis showed reduced binding of FOXA2 to the LINC00261 promoter in all 3 experiments performed, however, the difference did not reach statistical significance (* *p* < 0.05, *** *p* < 0.001; unpaired t-test).


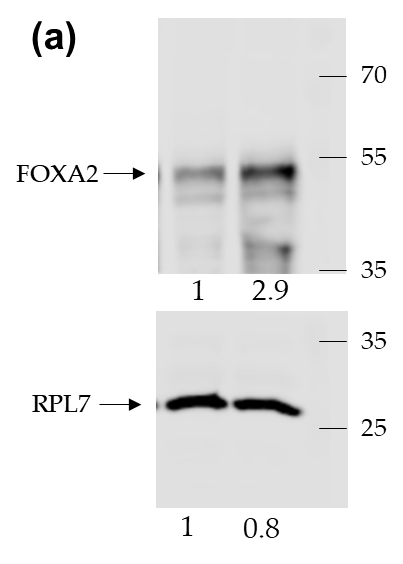

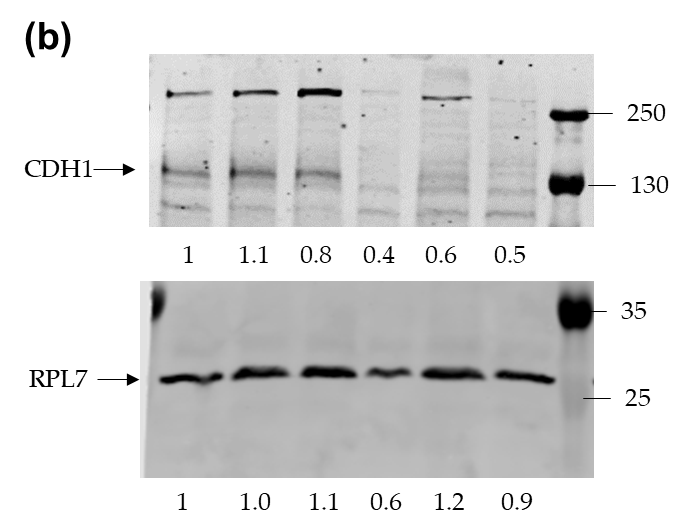


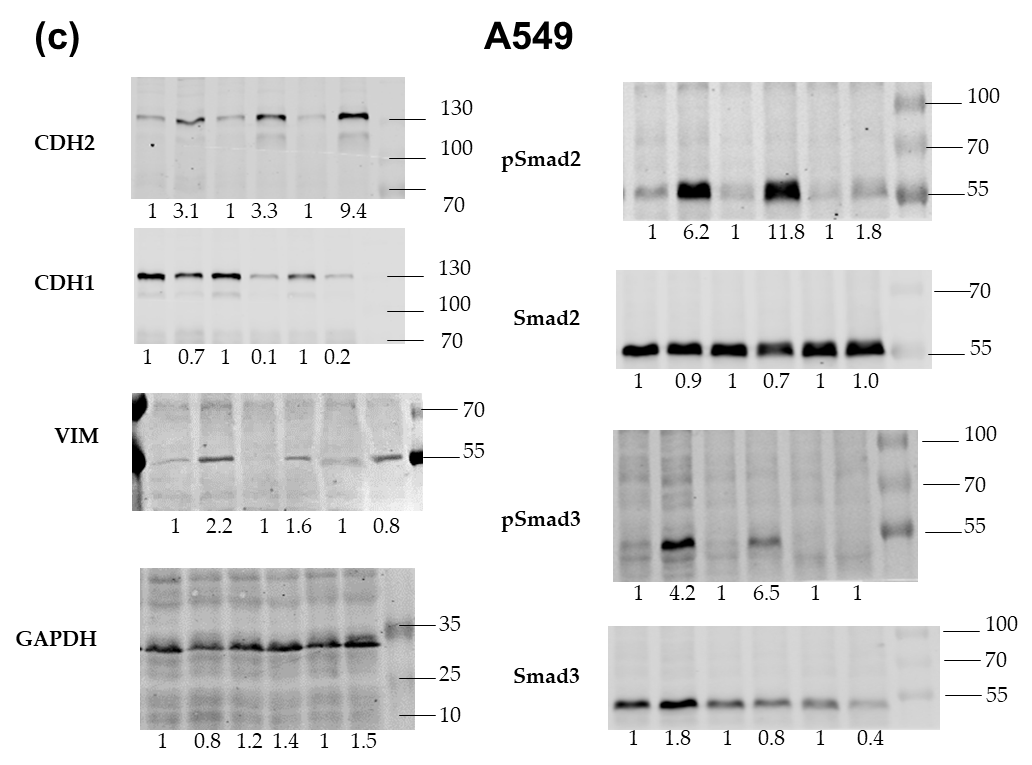


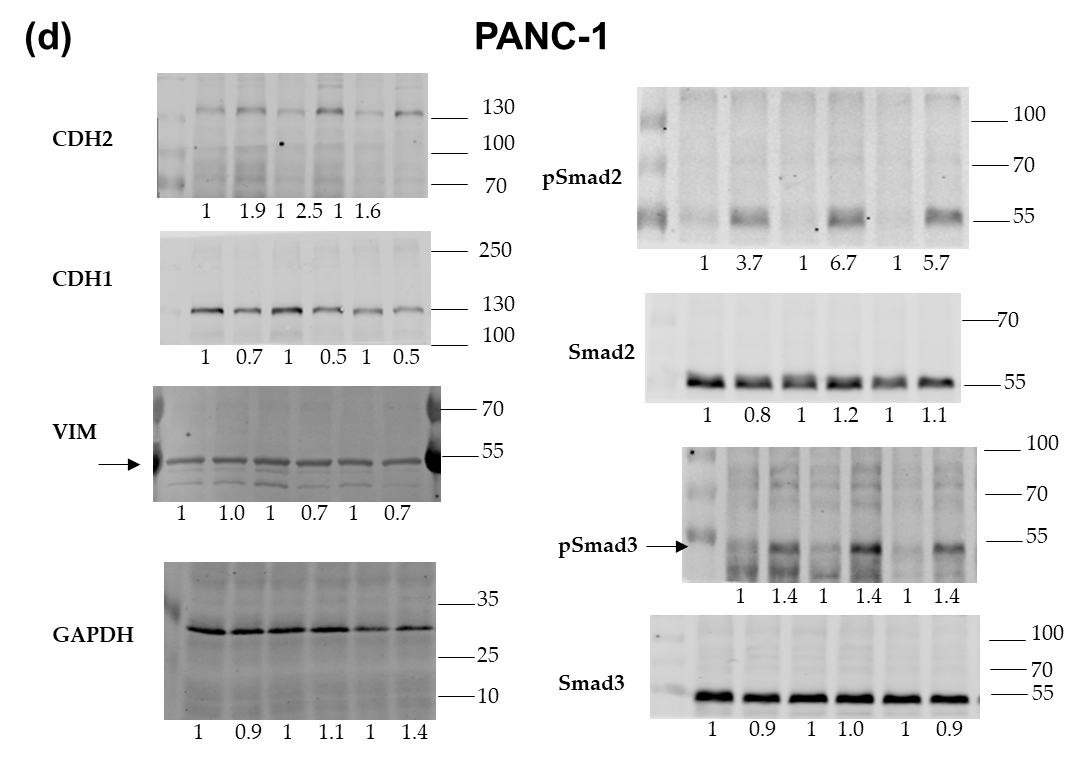


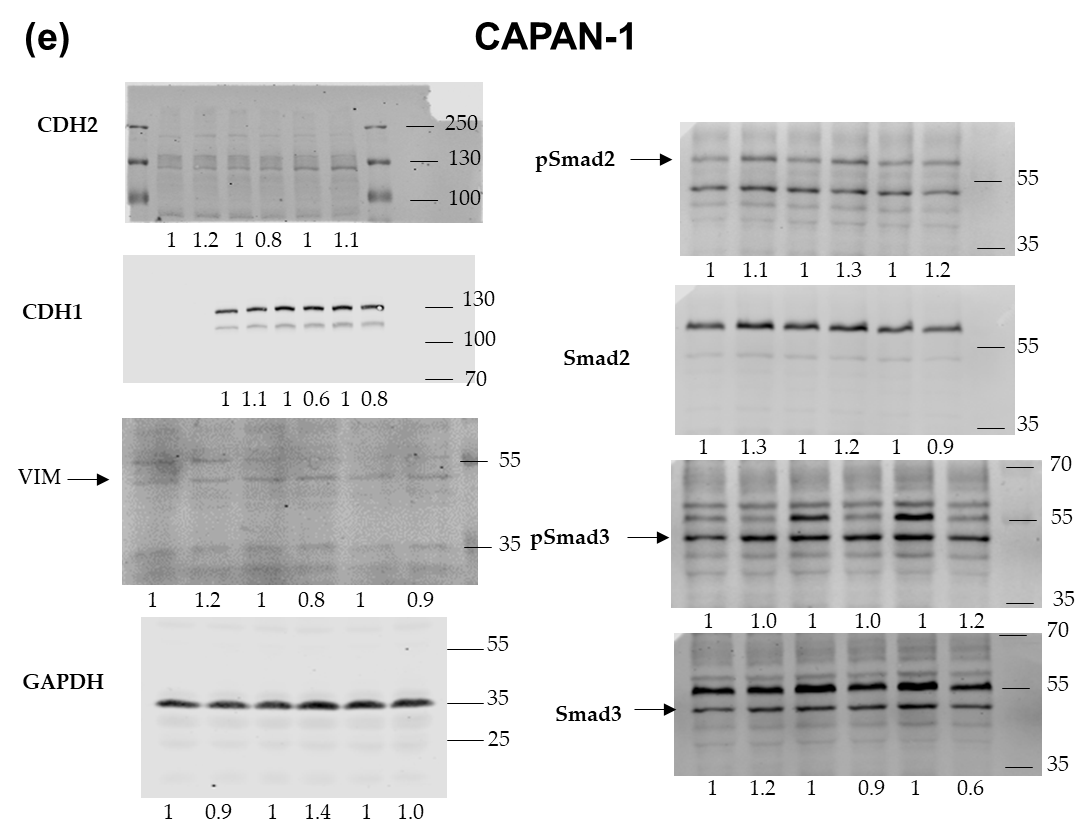


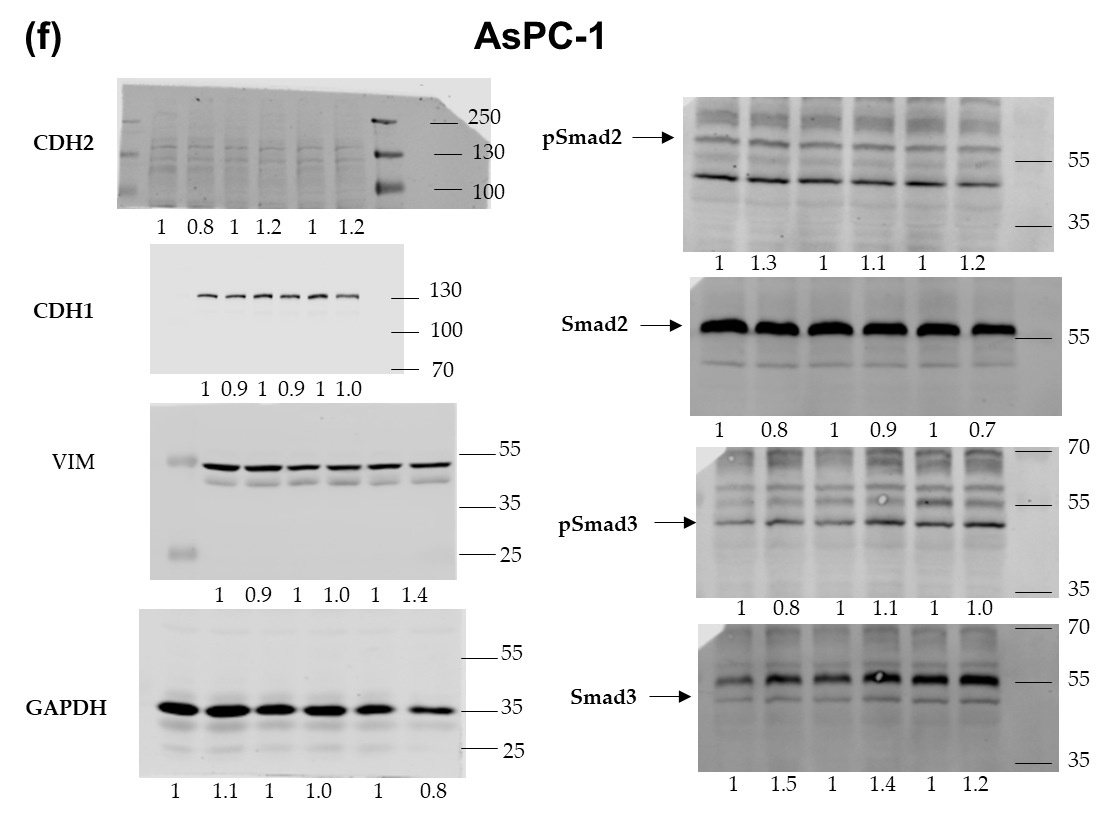


**Figure S5.** Uncropped blots with molecular weight markers and densitometry readings for Figure 3c (**a**), Figure 6b (**b**), Figure 4d (**c**,**d**) and Figure 4e (**e**,**f**).
